# Structure-based drug design targeting the cell membrane receptor GPBAR1: exploiting the bile acid scaffold towards selective agonism

**DOI:** 10.1038/srep16605

**Published:** 2015-11-16

**Authors:** Francesco Saverio Di Leva, Carmen Festa, Barbara Renga, Valentina Sepe, Ettore Novellino, Stefano Fiorucci, Angela Zampella, Vittorio Limongelli

**Affiliations:** 1 Department of Pharmacy, University of Naples “Federico II”, Via D. Montesano 49, I-80131 Naples, Italy; 2 Department of Surgery and Biomedical Sciences, Nuova Facoltà di Medicina, P.zza L. Severi, I-06132 Perugia, Italy; 3 Università della Svizzera Italiana (USI), Faculty of Informatics, Institute of Computational Science, via G. Buffi 13, CH-6900 Lugano, Switzerland

## Abstract

Bile acids can regulate nutrient metabolism through the activation of the cell membrane receptor GPBAR1 and the nuclear receptor FXR. Developing an exogenous control over these receptors represents an attractive strategy for the treatment of enterohepatic and metabolic disorders. A number of dual GPBAR1/FXR agonists are known, however their therapeutic use is limited by multiple unwanted effects due to activation of the diverse downstream signals controlled by the two receptors. On the other hand, designing selective GPBAR1 and FXR agonists is challenging since the two proteins share similar structural requisites for ligand binding. Here, taking advantage of our knowledge of the two targets, we have identified through a rational drug design study a series of amine lithocholic acid derivatives as selective GPBAR1 agonists. The presence of the 3α-NH_2_ group on the steroidal scaffold is responsible for the selectivity over FXR unveiling unprecedented structural insights into bile acid receptors activity modulation.

Bile acids (BAs), the end products of cholesterol metabolism, are amphipathic water-soluble compounds that regulate a variety of cellular functions. In the liver, cholesterol is first converted in primary bile acids, cholic acid (CA) and chenodeoxycholic acid (CDCA), which are in turn transformed by the intestinal microbiota in secondary bile acids, deoxycholic acid (DCA) and lithocholic acid (LCA). The latter derivatives can be finally modified in their glycine and taurine conjugates^1^. Both primary and secondary BAs take part to nutrients metabolism and cellular homeostasis through regulatory mechanisms that include the BAs interaction with nuclear (NRs) and cell membrane receptors^2^. Among bile acid NRs is the farnesoid X receptor (FXR) that is highly expressed in liver and in ileum epithelial cells, where it is generally activated by CDCA (Fig. 1)^3^,^4^. Upon ligand binding, FXR forms a heterodimer with the retinoid X receptor (RXR) that binds specific DNA sequences within the promoter regions of target genes. In such a way, FXR ligands regulate the transcription of proteins that control glucose, lipids and bile acid homeostasis^5^,^6^. An example of bile acid cell membrane receptors is instead the G-protein coupled bile acid receptor 1 (GPBAR1, TGR5, M-BAR1)^7^,^8^. The relevance and complexity of this receptor is reflected by its expression in heterogeneous cellular compartments such as gallbladder, liver, intestine, kidney, adipose tissue, skeletal muscle cells and macrophages/monocytes. Among the most potent natural agonists of GPBAR1 is the tauro-conjugated form of LCA, tauro-lithocolic acid (TLCA, **1**)^7^,^8^. The activation of this receptor increases intracellular levels of cAMP and protein kinase A functionality, allowing signaling transduction. Responses to GPBAR1 activation are tissue-specific and may include gallbladder relaxation, increased energy expenditure, improved intestinal motility, glucose metabolism and insulin sensitivity^1^,^9^,^10^. The latter two occur through the release of the glucagon-like peptide 1 (GLP-1) by intestinal L cells upon GPBAR1 activation^11^. Therefore, the exogenous regulation of this receptor represents an attractive strategy to treat severe enterohepatic and metabolic disorders such as nonalcoholic steatohepatitis (NASH), hypercholesterolaemia, hypertriglyceridaemia, and type 2 diabetes mellitus (T2DM)^2^,^12^,^13^.

The fact that activation of GPBAR1 and FXR can have similar effects acting on different downstream signals has prompted to develop dual GPBAR1/FXR agonists^2^,^13^,^14^,^15^. However, these compounds present a reduced therapeutic window, exposing patients to multiple unwanted effects due to activation of the diverse downstream signals controlled by the two receptors^6^,^16^. This leads to seek for selective ligands able to specifically activate only either one receptor. Indeed, selective GPBAR1 agonism allows controlling glucose metabolism through the release of glucagon-like peptide (GLP)-1 without affecting the FXR-related pathways. In order to design selective agonists, it is essential to know the BAs structural requisites to interact with GPBAR1 and FXR. Previous studies showed that the introduction of an ethyl group at C-6 on the CDCA ring B (6-ECDCA/OCA/INT-747)^17^, as well as the introduction on a sulfate group at position 24 or at position 23 on a shortened side chain (INT-767 and **2**)^14^,^15^, led to the development of potent dual agonists. Conversely, a marked selectivity toward GPBAR1 over FXR has been achieved through the methylation at C-23 position on the BA side chain (INT-777)^18^, and by stereochemical modification on ring B generating EUCDCOH, which is the first example of UDCA derivative substituted at C-6 with a β-oriented ethyl group^19^. Finally, independently from the functional group at C-24 on the side chain, the removal of the 3α-OH from the CDCA scaffold provides potent and selective FXR agonists^19^. In spite of these examples, a comprehensive understanding of the effects of BAs modifications on the activity and selectivity towards GPBAR1 and FXR is possible only if the binding mode of these compounds to the two receptors is elucidated. This is difficult because of the lack of the tridimensional structure of human GPBAR1 (hGPBAR1). We have lifted this limitation building the hGPBAR1 structure by homology modeling and performing a series of atomistic simulations and experiments on ligand/receptor binding using the hGPBAR1 model^15^ and the available rFXR crystal structure^20^. Our results are in fully agreement with previous mutagenesis data, reporting a decrease in the binding affinity for bile acid derivatives in the Asn93Ala, Glu169Ala and Tyr240Ala mutant forms of hGPBAR1^21^. In particular, we found that in GPBAR1 the 3α-OH on BA scaffold forms a stable H-bond interaction with the negatively charged side chain of Glu169 on the transmembrane helix-5 (TM-5) (Fig. 2A). At variance with GPBAR1, in FXR the 3α-OH group H-bonds with a positively charged residue, His444 on helix H12 (Fig. 2B). This ligand/receptor interaction stabilizes the cation−π interaction between His444 and Trp466 in the activation function-2 (AF-2) domain, which is essential for FXR activation^20^. The opposite charge on the interacting partner of 3α-OH group in the two receptors provides a hint to achieve a selective binding. In fact, the replacement of the 3α-OH on the BA scaffold with a positively charged group should lead to a selective GPBAR1 activation over FXR. Therefore, we have modified the scaffold of LCA by replacing the 3α-OH with a protonable -NH_2_ group and developed a small set of amine LCA derivatives (Fig. 3). The compounds have been synthesized and tested on GPBAR1 and FXR through specific pharmacological assays. These experiments demonstrate that through rational drug design we have achieved selectivity identifying the first amine LCA derivatives as selective GPBAR1 agonists. The binding mode of the most potent compound of the series to GPBAR1 has been also elucidated using docking simulations. Our findings open new routes to design selective GPBAR1 ligands and towards a rational modulation of BA receptors activity.

## Results

### Synthesis of amine LCA derivatives

**Figure 4:**
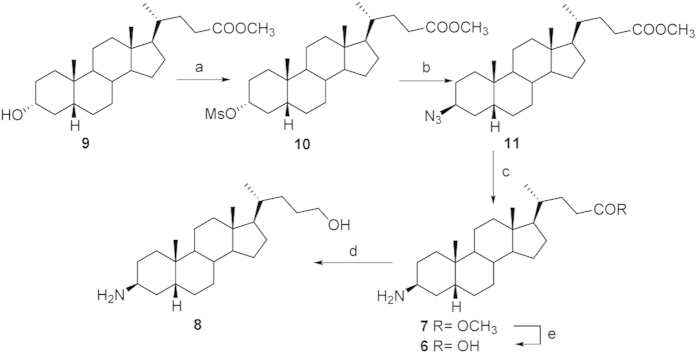
Preparation of β-amino LCA derivatives. Reagents and conditions: a) MsCl, TEA; ethyl ether, 53%; b) NaN_3_, DMSO, DMF, 150 °C, 64%; c) H_2_ (1 atm, Pd/C, THF/MeOH 1:1, 71%; d) LiBH_4_, MeOH dry, THF, 0 °C, 59%; e) NaOH 5% in MeOH/H_2_O 1:1 v/v, 62%.

**Figure 5:**
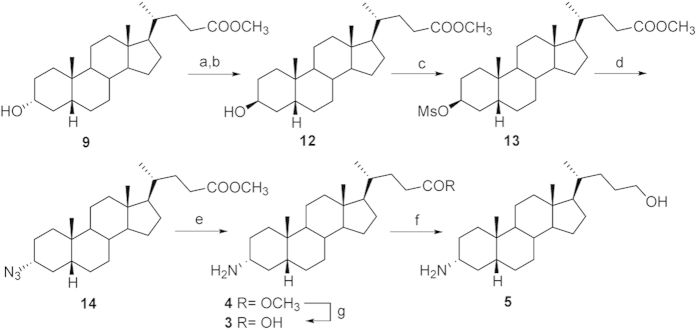
Preparation of α-amino LCA derivatives. Reagents and conditions: a) *p*-TsCl, pyridine, quantitative; b) CH_3_COOK, DMF; H_2_O 5:1, reflux, 81%; c) MsCl, TEA; ethyl ether, 67%; d) NaN_3_, DMSO, DMF, 150 °C, 67%; e) H_2_ (1 atm), Pd/C, THF/MeOH 1:1, 44%; f) LiBH_4_, MeOH dry, THF, 0 °C, 54%; g) NaOH 5% in MeOH/H_2_O 1:1 v/v, 42%.

Mesylation on LCA methyl ester **9** and subsequent treatment with NaN_3_ furnished the intermediate 3β-azido **11** (Fig. 4). Hydrogenation (H_2_, Pd/C) afforded methyl 3β-amino LCA **7** that was used as starting material for the preparation of **6** and **8** by methyl ester hydrolysis and methyl ester reduction, respectively (Fig. 4). The counterpart 3α-amino LCA derivatives were prepared following the synthetic protocol depicted in Fig. 5. Tosylation at C-3 hydroxyl group on LCA methyl ester followed by inversion of configuration with potassium acetate in DMF/H_2_O afforded the 3β-hydroxy derivative **12** in 81% over two steps, which was in turn transformed in the corresponding 3α-azido derivative **14** following the same synthetic protocol reported in Fig. 4. Hydrogenation at the azido group and elaboration of the functional group on the side chain afforded derivatives **3**–**5** in good chemical yield.

### Biological tests on amine LCA derivatives

**Figure 6:**
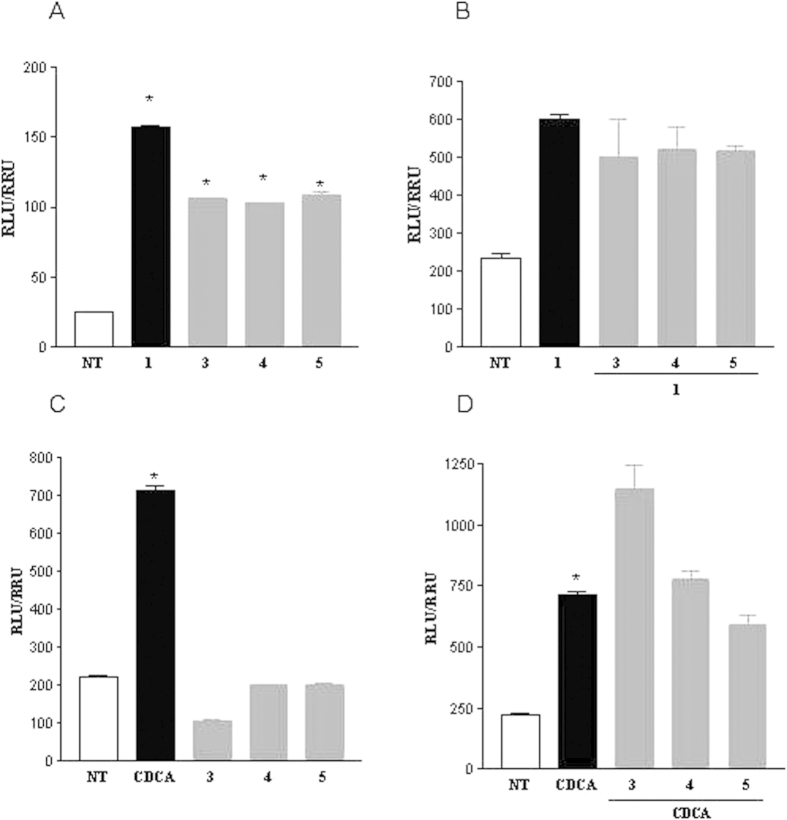
Transactivation assays on GPBAR1 (A,B) and FXR (C,D). Transactivation assays on GPBAR1. (**A**) HEK-293T cells were cotransfected with GPBAR1 and a reporter gene containing a cAMP responsive element in front of the luciferase gene. Cells were stimulated 18 h with compounds **3**, **4**, and **5** at 10 μM and TLCA (10 μM) was used as a positive control. (**B**) To evaluate antagonistic activity of compounds **3**, **4** and **5** cells were stimulated 18 h with 50 μM **3**, **4** and **5** in the presence of TLCA (10 μM). Results are expressed as mean ± standard error; *p < 0.05 versus not treated cells (NT). (**C,D**) Transactivation assays on FXR. (**C**) HepG2 cells were transfected with pSG5-FXR, pSG5-RXR, p(hsp27)TKLUC and pGL4.70 vectors. Cells were stimulated 18 h with **3**, **4** and **5** at 10 μM and CDCA (10 μM) was used as a positive control. (**D**) Cells were stimulated for 18 h with 50 μM **3**, **4** and **5** in the presence of CDCA (10 μM). Results are expressed as mean ± standard error; *p < 0.05 versus not treated cells (NT).

**Figure 7:**
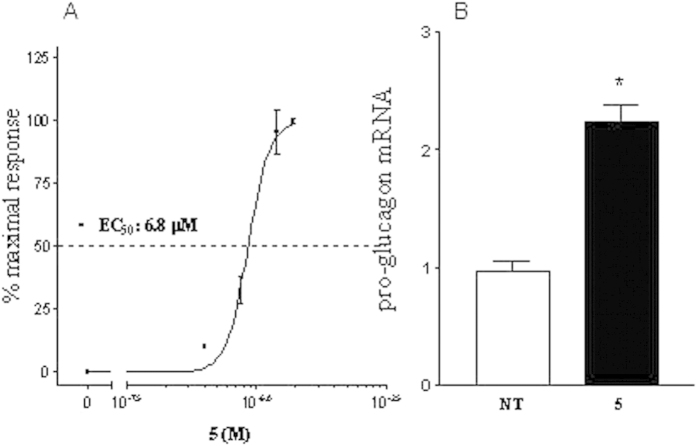
Concentration-response curve on GPBAR1 and RT-PCR experiments on GLUTAg cells. (**A**) Concentration-response curve on GPBAR1 activation by compound **5**. (**B**) Effect of **5** on relative mRNA expression of pro-glucagon in GLUTAg cells left untreated (NT) or stimulated with 10 μM compound **5**. Values are normalized relative to GAPDH mRNA and are expressed relative to those of nontreated cells (NT), which are arbitrarily set to 1: *p < 0.05 vs NT.

Derivatives **3**–**8** were tested for their activity on FXR and GPBAR1. For this purpose, we performed a luciferase reporter assay using HepG2 and HEK-293T cells transfected with FXR and GPBAR1, respectively (Fig. 6). Unfortunately compounds **6**–**8**, featuring the amino group at C-3 in β configuration were proved to be cytotoxic on HEK-293T cells at 10 μM (data not shown). Of interest, the results shown in Fig. 6 demonstrate that all 3α-amino LCA derivatives generated in this study are agonists of GPBAR1 (Fig. 6A). Indeed, we found that the substitution of the hydroxyl group at C-3 on LCA scaffold with a α-amino group produces selective GPBAR1 agonists, devoid of FXR agonist properties (Fig. 6C). On the other hand, none of the tested compounds turned out to be GPBAR1 antagonist (Fig. 6B). However, when compound **3** was tested at 50 μM in the presence of CDCA (Fig. 6D), the relative luciferase/renilla units ratio RLU/RRU was higher than that of CDCA, indicating that **3** might be also an FXR modulator. Overall, LCA 3α-amino derivatives thus represent promising templates for generating selective GPBAR1 modulators. The potency of compound **5**, a selected member of our set of LCA derivatives, was further investigated by a detailed measurement of concentration-response curve on GPBAR1. As shown in Fig. 7A, compound **5** effectively induced GPBAR1 transactivation in a concentration-dependent manner, with a relative EC_50_ of 6.8 μM. Moreover, the agonism of **5** on GPBAR1 was also substantiated by RT-PCR experiments where **5** was effective in inducing the expression of pro-glucagon mRNA in GLUTAg cells, an intestinal endocrine cell line (Fig. 7B).

### Computational studies

**Figure 8:**
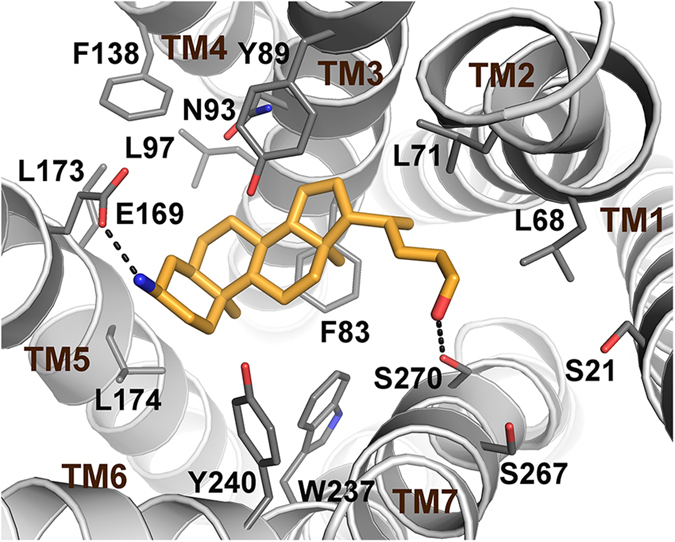
Predicted binding mode of 5 in hGPBAR1. **5** is shown as cyan sticks. GPBAR1 is shown as grey cartoons and sticks. GPBAR1 extracellular loops and nonpolar hydrogens are omitted for clarity.

To elucidate the binding mode of the amine LCA derivatives to GPBAR1 molecular docking studies were performed. In particular, the most potent compound of the series, compound **5**, was docked in the homology model of hGPBAR1 that we have previously reported^15^. In the best-scored docking pose, **5** interacts with GPBAR1 similarly to derivative **2** (Fig. 8). In particular, the steroidal scaffold of **5** lodges in a hydrophobic cleft where it engages favorable interactions with residues such Leu71, Phe83, Leu174, and Trp237. The positively charged α-amine group of **5** forms charge-enforced H-bonds with the side chain of Glu169 on TM5 occupying the binding site similarly to the 3α-OH of **5**. On the other side, the ligand flexible side chain points toward TM helices 1, 2, and 7, where the alcohol group can form H-bond interactions with the Ser270 side chain. Our calculations indicate that in the ligand side chain diverse functional groups able to interact with Ser21, Ser267 and Ser270 can be considered. This is confirmed by the pharmacological assays showing that compounds **3**, **4** and **5**, with a carboxylate, ester and alcohol side chain, respectively, retain GPBAR1 agonist activity, however bearing side chains with different steric and electrostatic properties.

## Discussion

In the last decade the BA receptors GPBAR1 and FXR have emerged as prominent targets for treatment of several lipid and glucose disorders, including type 2 diabetes mellitus^1^,^2^. With the intention of achieving a synergic effect, medicinal chemists have devoted many efforts to develop dual GPBAR1/FXR agonists^2^,^13^,^14^,^15^. Unfortunately, the therapeutic use of these compounds is limited by multiple unwanted effects due to activation of the diverse downstream signals controlled by the two receptors^6^,^16^. As a consequence, efforts have been shifted towards the development of receptor-selective ligands. This goal is however hard to pursue since the two receptors share similar structural requisites for ligand binding, thus hampering rational drug design. Elucidating the binding mode of diverse BAs to GPBAR1 and FXR is the first necessary step towards a full understanding of the molecular bases for a selective interaction. We contributed in this context performing extensive simulations and experiments on binding of a series of BA derivatives to the two targets^15^,^22^. Our studies have been instrumental and inspiring for drug design leading us to develop and synthesize a small set of amine LCA derivatives (**3**–**8**). Three compounds of the series (**3**–**5**) turned out as selective GPBAR1 agonists, showing EC_50_ values in the low micromolar range. To our knowledge, these are the first examples of amine BA derivatives active on GPBAR1, thus providing new possibilities to achieve selective ligands with improved drug-like properties and a rational modulation of BA receptors activity.

## Methods

### Molecular Docking

The Glide (version 6.7)^23^ and AutoDock4.2 (AD4)^24^ software packages were used to perform molecular docking calculations in the three-dimensional model of hGPBAR1. Ligands and receptor structures were prepared as described in a previous paper^22^. In Glide, a box centered on the GPBAR1 binding cavity was created; the Cartesian coordinates of the box, X, Y, and Z length were all set to 24.74, 26.74, 22.74 Å, resepctively. The standard precision (SP) mode of the GlideScore function was used to score the predicted binding poses. In AD4, grid points of 50 × 45 × 30 with a 0.375 Å spacing were calculated around the binding cavity using AutoGrid4.2. Thus, 100 separate docking calculations were performed for each run. Each docking run consisted of 10 million energy evaluations using the Lamarckian genetic algorithm local search (GALS) method. Otherwise default docking parameters were applied. Docking conformations were clustered on the basis of their rmsd (tolerance = 2.0 Å) and were ranked based on the AutoDock scoring function^25^. The choice of combining two docking software was motivated by both the different algorithm used for ligand conformational analysis and the diverse scoring functions. The binding mode shown in Fig. 8 corresponds to the Glide best-ranked docking pose (see Table S1) and is representative of the conformation cluster family among the best-scored ones by AD4 that is in agreement with previously reported BA binding modes in hGPBAR1 and mutagenesis experiments^15^,^21^,^22^.

All the residue labels were taken from crystal structure of rFXR-LBD with PDB ID 1osv and the wild-type amino acidic sequence of human GPBAR1.

All figures were rendered using PyMOL (http://www.pymol.org).

### Chemistry

Specific rotations were measured on a Jasco P-2000 polarimeter. High-resolution ESI-MS spectra were performed with a Micromass Q-TOF mass spectrometer. NMR spectra were obtained on Varian Inova 400, 500 and 700 NMR spectrometers (^1^H at 400, 500 and 700 MHz, ^13^C at 100, 125 and 175 MHz, respectively) equipped with a SUN microsystem ultra5 hardware and recorded in CD_3_OD (δ_H_ = 3.31 and δ_C_ = 49.0 ppm) and CDCl_3_ (δ_H_ = 7.26 and δ_C_ = 77.0 ppm). All of the detected signals were in accordance with the proposed structures. Coupling constants (*J* values) are given in Hertz (Hz), and chemical shifts (δ) are reported in ppm and referred to CHD_2_OD and CHCl_3_ as internal standards. Spin multiplicities are given as s (singlet), br s (broad singlet), d (doublet), t (triplet) or m (multiplet).

HPLC was performed with a Waters Model 510 pump equipped with Waters Rheodine injector and a differential refractometer, model 401. Reaction progress was monitored via thin-layer chromatography (TLC) on Alugram silica gel G/UV254 plates. Silica gel MN Kieselgel 60 (70–230 mesh) from Macherey-Nagel Company was used for column chromatography. All chemicals were obtained from Sigma- Aldrich, Inc.

Solvents and reagents were used as supplied from commercial sources with the following exceptions. Hexane, ethyl acetate, chloroform, dichloromethane, tetrahydrofuran and triethylamine were distilled from calcium hydride immediately prior to use. Methanol was dried from magnesium methoxide as follow. Magnesium turnings (5 g) and iodine (0.5 g) were refluxed in a small (50–100 mL) quantity of methanol until all of the magnesium has reacted. The mixture was diluted (up to 1 L) with reagent grade methanol, refluxed for 2–3 h then distilled under nitrogen. All reactions were carried out under argon atmosphere using flame-dried glassware.

The purity of tested compounds was determined to be always greater than 95% by analytical HPLC analysis using a Nucleodur 100-5 C18 (5 μm; 4.6 mm i.d. × 250 mm) column eluting with the solvent system (flow rate 1 mL/min) reported below in the section corresponding to each individual compound.

### Synthetic procedures

See the Supporting Information.

### Cell culture

HepG2, an immortalized epatocarcinoma cell line, was cultured and maintained at 37 °C and 5% CO_2_ in E-MEM additioned with 10% FBS, 1% glutamine and 1% penicillin/streptomycin. HEK293T and Glutag cells were cultured and maintained at 37 °C and 5% CO_2_ in D-MEM with 10% FBS, 1% glutamine and 1% penicillin/streptomycin.

### Luciferase reporter gene assay

To evaluate FXR mediated transactivation, HepG2 cells were transfected with 100 ng of pSG5-FXR, 100 ng of pSG5-RXR, 200 ng of the reporter vector p(hsp27)-TK-LUC containing the FXR response element IR1 cloned from the promoter of heat shock protein 27 (hsp27) and with 100 ng of pGL4.70 (Promega), a vector encoding the human Renilla gene. To evaluate GPBAR1 mediated transactivation, HEK-293T cells were transfected with 200 ng of pGL4.29 (Promega), a reporter vector containing a cAMP response element (CRE) that drives the transcription of the luciferase reporter gene luc2P, with 100 ng of pCMVSPORT6-human GPBAR1, and with 100 ng of pGL4.70. At 24 h post-transfection, cells were stimulated 18 h with 10 μM TLCA, CDCA, compounds **3**, **4**, **5**. In another experimental setting at 24 h post-transfection cells were stimulated with 50 μM **3**, **4**, **5** in combination of 10 μM CDCA or TLCA. To calculate the EC_50_ of **5** versus GPBAR1 a dose response curve was performed in HEK-293T transfected as described above and stimulated 18 h with 1, 5, 25 and 50 μM compound **5**. After treatments, 10 μL of cellular lysates were read using Dual Luciferase Reporter Assay System (Promega Italia srl, Milan, Italy) according manufacturer specifications using the Glomax 20/20 luminometer (Promega Italia srl, Milan, Italy). Luciferase activities were assayed and normalized with Renilla activities.

### Real-Time PCR

Total RNA was isolated from Glutag cells left untreated or stimulated 18 h with 10 μM compound **5** using the TRIzol reagent according to the manufacturer’s specifications (Invitrogen). One microgram of purified RNA was treated with DNase-I and reverse transcribed with Superscript II (Invitrogen). For Real Time PCR, 10 ng template was dissolved in 25 μL containing 200 nmol/L of each primer and 12.5 μL of 2X SYBR FAST Universal ready mix (Invitrogen). All reactions were performed in triplicate, and the thermal cycling conditions were as follows: 2 min at 95 °C, followed by 40 cycles of 95 °C for 20 s and 60 °C for 30 s in iCycler iQ instrument (Biorad). The relative mRNA expression was calculated and expressed as 2−(ΔΔCt). Forward and reverse primer sequences were the following: mouse GAPDH, ctgagtatgtcgtggagtctac and gttggtggtgcaggatgcattg; mouse Pro-glucagon, tgaagacaaacgccactcac and caatgttgttccggttcctc.

## Additional Information

**How to cite this article**: Di Leva, F. S. *et al.* Structure-based drug design targeting the cell membrane receptor GPBAR1: exploiting the bile acid scaffold towards selective agonism. *Sci. Rep.* **5**, 16605; doi: 10.1038/srep16605 (2015).

## Supplementary Material

Supplementary Information

## Figures and Tables

**Figure 1:**
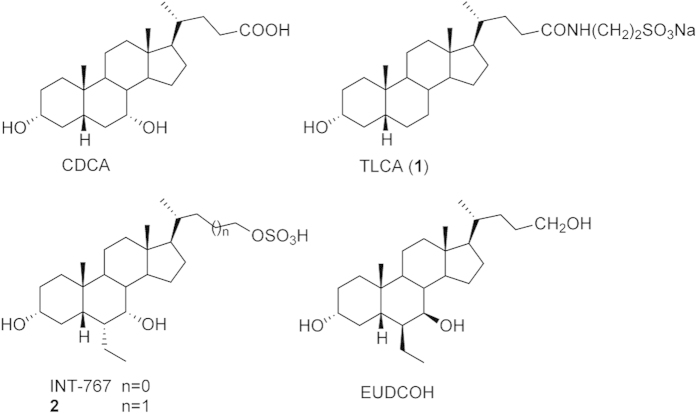
Natural and semi-synthetic GPBAR1 and FXR agonists. CDCA and TLCA, endogenous activators of FXR and GPBAR1, respectively. INT-767 and compound **2**, potent dual FXR/GPBAR1 agonists and EUDCOH, a selective GPBAR1 agonist.

**Figure 2:**
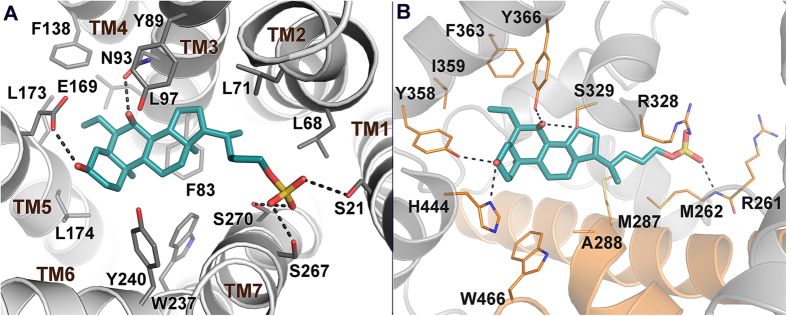
Predicted binding mode of 2 in hGPBAR1 (A) and rFXR (B). **2** is shown as cyan sticks. The receptors are shown as gray and orange (helices H3, H4, and H12 in FXR) cartoons and sticks. Extracellular loops of GPBAR1 and nonpolar hydrogens are omitted for clarity.

**Figure 3:**
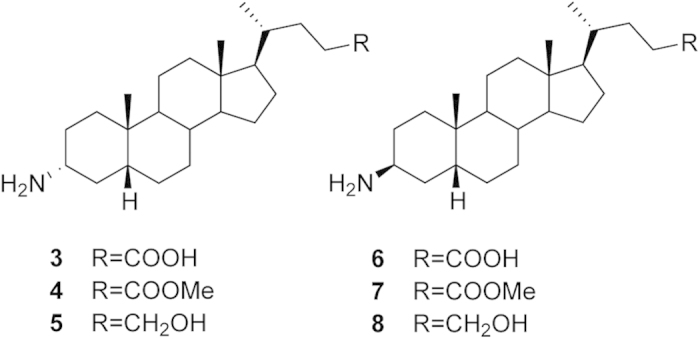
α-Amino LCA (3–5) and β-amino LCA (6–8) derivatives synthesized in this study.
