# Supplementary material for: Structure-based drug design targeting the cell membrane receptor GPBAR1: exploiting the bile acid scaffold towards selective agonism
Source: Sci Rep. 2015 Nov 16;5:16605. doi: 10.1038/srep16605 (PMC4645117; doi:10.1038/srep16605)
Supplement: Supplementary Information [file srep16605-s1.doc]

**Supporting Information**

Design, synthesis and biological evaluation of amine bile acid derivatives as GPBAR1 selective agonists

Francesco Saverio Di Leva,**1‡** Carmen Festa,**1‡** Barbara Renga,2**‡** Valentina Sepe,**1** Ettore Novellino,**1** Stefano Fiorucci,2* Angela Zampella,1* and Vittorio Limongelli1,3*

1Department of Pharmacy, University of Naples “Federico II”, Via D. Montesano 49, I-80131 Naples, Italy.

2Department of Surgery and Biomedical Sciences, Nuova Facoltà di Medicina, P.zza L. Severi, I-06132 Perugia, Italy.

3Università della Svizzera Italiana (USI), Faculty of Informatics, Institute of Computational Science, via G. Buffi 13, CH-6900 Lugano, Switzerland

Table of contents S1

Synthetic procedures S2

1H-NMR spectrum of compound **3** S8

13C NMR spectrum of compound **3** S8

1H-NMR spectrum of compound **4** S9

13C NMR spectrum of compound **4** S9

1H-NMR spectrum of compound **5** S10

13C NMR spectrum of compound **5** S10

1H-NMR spectrum of compound **6** S11

13C NMR spectrum of compound **6** S11

1H-NMR spectrum of compound **7** S12

13C NMR spectrum of compound **7** S12

1H-NMR spectrum of compound **8** S13

13C NMR spectrum of compound **8** S13

Table S1 S14

**Synthetic procedures.**

**Methyl 3α-hydroxy-5β-cholan-24-oate (9).** LCA (500 mg, 1.33 mmol) was dissolved in 5 mL of dry methanol and treated with *p-*toluenesulfonic acid in catalytic amount. The solution was left to stand at room temperature for 5 h. The mixture was quenched by addition until the neutrality of NaHCO3 saturated solution. Most of the solvent was evaporated, and the residue was extracted with EtOAc. The combined extract was washed with brine, dried with Na2SO4, and evaporated to give the corresponding methyl ester **9** as amorphous solid (550 mg, quantitative yield). [α]25D=+19.3 (*c* 0.37, CH3OH); selected 1H NMR (400 MHz CDCl3):  3.65 (3H, s), 3.62 (1H,m), 2.42 (1H, m), 2.27 (1H, m), 0.93 (3H, d, ovl), 0.92 (3H, s), 0.64 (3H, s). HRMS-ESI *m/z* 391.3219 [M+H+], C24H43O3 requires 391.3212.

**Methyl 3β-hydroxy-5β-cholan-24-oate (12)**. To a solution of LCA methyl ester **9** (300 mg, 0.77 mmol) in dry pyridine (5 mL), tosyl chloride (734 mg, 3.85 mmol) was added, and the mixture was stirred at room temperature for 2 h. It was poured into cold water (10 mL) and extracted with CH2Cl2 (3 × 10 mL). The combined organic layer was washed with saturated NaHCO3 solution (10 mL), and water (10 mL), and then dried over anhydrous MgSO4 and evaporated *in vacuo* to give 430 mg of methyl 3α-tosyloxy-5β-cholan-24-oate (quantitative yield) in the form of colourless needles, that was subjected to next step without any purification. [α]D25= +59.5 (*c* 0.02, CH3OH); selected 1H NMR (500 MHz, CDCl3): δH 7.77 (2H, d, *J* = 8.2 Hz), 7.31 (2H, d, *J* = 8.2 Hz), 4.43 (1H, m), 3.64 (3H, s), 2.32 (1H, m), 2.19 (1H, m), 0.88 (3H, d, *J* = 6.6 Hz), 0.86 (3H, s), 0.60 (3H, s); HR ESIMS *m/z*545.3309 [M + H]+, C32H49O5S requires 545.3301.

A solution of methyl 3α-tosyloxy-5β-cholan-24-oate (430 mg, 0.79 mmol) and CH3COOK (78 mg, 0.79 mmol) dissolved in water (2 mL) and N,N’-dimethylformamide (DMF, 8 mL) was refluxed for 5 h. The solution was cooled at room temperature and then ethyl acetate and water were added. The separated aqueous phase was extracted with ethyl acetate (3 ×30 mL). The combined organic phases were washed with water, dried (Na2SO4) and evaporated to dryness to give 600 mg of mixture. Purification by silica gel (hexane-ethyl acetate 8:2 and 0.5% TEA) gave compound **12** (250 mg, 81%). [α]D25= +19.1 (*c* 0.12, CH3OH); selected 1H NMR (500 MHz, CDCl3): δH 4.10 (1H, br s), 3.66 (3H, s), 2.34 (1H, m), 2.22 (1H, m), 0.96 (3H, s), 0.91 (3H, d, *J* = 6.5 Hz), 0.65 (3H, s); HR ESIMS *m/z* 391.3220 [M + H]+, C25H43O3 requires 391.3212.

**Methyl 3β-mesyloxy-5β-cholan-24-oate (13)**. To a solution of **12** (250 mg, 0.64 mmol) in dry ethyl ether (3 mL), mesyl chloride (246 μL, 3.2 mmol) and TEA (534 μL, 3.8 mmol) was added, and the mixture was stirred at -10°C for 3 h. It was poured into saturated NaHCO3 solution (10 mL) and extracted with ethyl ether (3 × 10 mL). The combined organic layer was washed with water (10 mL), and then dried over anhydrous MgSO4 and evaporated *in vacuo* to give 200 mg of methyl 3β-mesyloxy-5β-cholan-24-oate **13** (67% yield), that was subjected to next step without any purification. [α]D25= +11.0 (*c* 0.33, CH3OH); selected 1H NMR (400 MHz, CDCl3): δH 5.03 (1H, br s), 3.64 (3H, s), 2.96 (3H, s), 2.33 (1H, m), 2.20 (1H, m), 0.96 (3H, s), 0.90 (3H, d, *J* = 6.3 Hz), 0.64 (3H, s); 13C NMR (125 MHz, CDCl3): δC 174.7, 80.5, 56.2, 55.6, 51.2, 49.8, 42.4, 39.7, 38.2, 36.5, 35.2, 35.0, 34.4, 31.4, 30.7 (2C), 29.7, 27.8, 25.9 (2C), 25.7, 23.8, 23.3, 20.7, 17.9, 11.7; HR ESIMS *m/z* 469.2993 [M + H]+, C26H45O5S requires 469.2988.

**Methyl 3α-azido-5β-cholan-24-oate (14).** Compound **13** (200 mg, 0.43 mmol) was dissolved in dry DMSO (5 ml) and sodium azide (278 mg, 4.3 mmol) was added. The mixture was stirred vigorously at 150 °C for 24 hours then partitioned between water and ethyl acetate (10 ml). The organic phases were dried (MgSO4), filtered and concentrated in vacuo to give 300 mg of mixture. Purification by silica gel (hexane-ethyl acetate 9:1 and 0.5% TEA) gave compound **14** (120 mg, 67%). (.[α]D25= +13.4 (*c* 0.07, CH3OH); selected 1H NMR (400 MHz, CD3OD): δH 3.65 (3H, s), 3.37 (1H, m), 2.35 (3H, s), 2.24 (1H, m), 0.97 (3H, s), 0.94 (3H, d, *J* = 6.5 Hz), 0.69 (3H, s); 13C NMR (105 MHz, CD3OD) δC 174.2, 61.6, 55.6, 55.3, 50.9, 42.1, 41.7, 40.4, 39.8, 39.5, 35.1, 34.9, 34.7, 34.0, 31.8, 30.4, 27.6, 26.5, 26.2, 25.7, 23.6, 22.9, 20.2, 17.7, 11.5; HR ESIMS *m/z* 416.282 [M + H]+, C25H42O2N3 requires 416.3277.

**Methyl 3α-ammino-5β-cholan-24-oate (4).** An oven-dried 10 mL flask was charged with 10% palladium on carbon (5 mg) and compound **14** (120 mg, 0.29 mmol) and the flask was evacuated and flushed with argon. Absolute methanol (2 mL) and dry THF (2 mL) were added, and the flask was flushed with hydrogen. The reaction was stirred at room temperature under H2 (1 atm) over night. The mixture was filtered through celite, and the recovered filtrate was concentrated to give 95 mg of crude product, that was further purified by HPLC on a Nucleodur 100-5 C18 (5 μm; 5 mm i.d. x 250 mm) with MeOH:H2O (70:30) as eluent (flow rate 1 mL/min), to give 50 mg of compound **4** (44%, tR= 11.0 min). [α]D25= +22 (*c* 0.15, CH3OH); selected 1H NMR (400 MHz, CD3OD): δH 3.64 (3H, s), 3.03 (1H, m), 2.35 (3H, s), 2.25 (1H, m), 0.99 (3H, s), 0.94 (3H, d, *J* = 6.7 Hz), 0.70 (3H, s); 13C NMR (100 MHz, CD3OD): C 176.4, 57.9, 57.5, 52.3, 52.0, 43.9, 43.2, 41.8, 41.5, 37.1, 36.7, 36.0, 35.6, 32.4, 32.2, 31.9, 29.2, 28.0, 27.4, 26.7, 25.2, 23.7, 21.9, 18.7, 12.4; HR ESIMS *m/z* 390.3376 [M + H]+, C25H44O2N requires 390.3372.

**Methyl 3α-ammino-5β-cholan-24-oic acid (3).** Compound **4** (20 mg, 51.4 x 10-3 mmol) was hydrolyzed with a methanol solution of sodium hydroxide (5%, 5 mL) in H2O (1 mL) overnight under reflux. The resulting solution was then concentrated under vacuum, diluted with water, acidified with HCl 6 N and extracted with ethyl acetate (3 x 10 mL). The collected organic phases were washed with brine, dried over Na2SO4 anhydrous and evaporated under reduced pressure. HPLC purification on a Nucleodur 100-5 C18 (5 μm; 5 mm i.d. x 250 mm) with MeOH/H2O (70:30) with 0.1% TFA as eluent (flow rate 1 mL/min), gave8 mg of **5** as white solid (42%, tR= 21 min). [α]D25= +10.0 (*c* 0.19, CH3OH); selected 1H NMR (700 MHz, CD3OD): δH 3.11 (1H, m), 2.32 (1H, m), 2.19 (1H, m), 1.00 (3H, s), 0.95 (3H, d, *J* = 6.5 Hz), 0.70 (3H, s); 13C NMR (175 MHz, CD3OD): C 170.5, 57.9, 57.5, 52.3, 43.9, 43.2, 41.8, 41.4, 37.1, 36.7, 36.0, 35.6, 32.4, 32.3, 29.2, 27.9, 27.4, 26.6 (2C), 25.2, 23.7, 21.9, 18.7, 12.4; HR ESIMS *m/z* 374.3062 [M - H]­, C24H40O2N requires 374.3059.

**Methyl 3α-ammino-5β-cholan-24-ol (5).** To a solution of methyl ester **4** (20 mg, 51.4 x 10-3 mmol) in dry THF (5 mL), at 0 °C dry methanol (15 μL, 0.36 mmol) and LiBH4 (180 μL, 2 M in THF, 0.36 mmol) was added. The resulting mixture was stirred for 2 h at 0 °C. The mixture was quenched by addition NaOH 1 M (10 μL) and then ethyl acetate. The organic phase was washed with water, dried (Na2SO4) and concentrated. HPLC purification on a Nucleodur 100-5 C18 (5 μm; 10 mm i.d. x 250 mm) with MeOH/H2O (65:35) as eluent (flow rate 1 mL/min), gave 10 mg of compound **5** as a white solid (54%, tR= 8.5 min). [α]D25= +38.8 (*c* 0.03, CH3OH); selected 1H NMR (700 MHz, CD3OD): δH 3.50 (2H, m), 3.06 (1H, m), 1.00 (3H, s), 0.95 (3H, d, *J* = 6.5 Hz), 0.71 (3H, s). 13C NMR (100 MHz CD3OD): C 63.6, 57.9, 57.6, 52.3, 43.9, 43.4, 41.8, 41.5, 37.1, 37.0, 36.2, 35.6, 33.2, 33.0, 30.2, 29.4, 28.0, 27.5, 27.2, 25.3, 23.8, 21.9, 19.2, 12.4; HR ESIMS *m/z* 362.3430 [M + H]+, C24H44ON requires 362.3423.

**Methyl 3α-mesyloxy-5β-cholan-24-oate (10)**. To a solution of LCA methyl ester **9** (250 mg, 0.64 mmol) in dry ethyl ether (10 mL), mesyl chloride (247 μL, 3.2 mmol) and TEA (535 μL, 3.8 mmol) was added, and the mixture was stirred at -10°C for 1 h. It was poured into saturated NaHCO3 solution (10 mL) and extracted with ethyl ether (3 × 10 mL). The combined organic layer was washed with water (10 mL), and then dried over anhydrous MgSO4 and evaporated *in vacuo* to give 160 mg of methyl 3α-mesyloxy-5β-cholan-24-oate **10** (53% yield), that was subjected to next step without any purification. [α]D25= +39.8 (*c* 0.08, CH3OH); selected 1H NMR (400 MHz, CDCl3): δH 4.63 (1H, m), 3.64 (3H, s), 2.98 (3H, s), 2.33 (1H, m), 2.20 (1H, m), 0.92 (3H, s), 0.90 (3H, d, *J* = 6.6 Hz), 0.63 (3H, s); HR ESIMS *m/z* 469.2991 [M + H]+, C26H45O5S requires 469.2988.

**Methyl 3β-azido-5β-cholan-24-oate (11).** Compound **10** (160 mg, 0.34 mmol) was dissolved in dry DMSO (5 ml) and sodium azide (221 mg, 3.4 mmol) was added. The mixture was stirred vigorously at 150 °C for 24 hours then partitioned between water and ethyl acetate (10 ml). The organic phases were dried (MgSO4), filtered and concentrated *in vacuo* to give 230 mg of mixture. Purification by silica gel (hexane-ethyl acetate 9:1 and 0.5% TEA) gave compound **11** (90 mg, 64%). [α]D25= +6.3 (*c* 0.53, CH3OH); selected 1H NMR (500 MHz, CD3OD): δH 3.91 (1H, br s), 3.63 (3H, s), 2.30 (1H, m), 2.18 (1H, m), 0.92 (3H, s), 0.88 (3H, d, *J* = 6.4 Hz), 0.62 (3H, s); 13C NMR (100 MHz, CD3OD): δC 174.6, 60.2, 57.8, 57.4, 51.5, 43.9, 41.5, 41.4, 38.8, 37.1, 36.7, 36.0, 32.3, 32.0, 31.8, 31.3, 29.2, 27.7, 27.4, 25.6, 25.3, 24.4, 22.1, 18.7, 12.5; HR ESIMS *m/z* 416.3279 [M + H]+, C25H42O2N3 requires 416.3277.

**Methyl 3β-ammino-5β-cholan-24-oate (7).** An oven-dried 10 mL flask was charged with 10% palladium on carbon (5 mg) and compound **11** (90 mg, 0.21 mmol) and the flask was evacuated and flushed with argon. Absolute methanol (5 mL) and dry THF (5 mL) were added, and the flask was flushed with hydrogen. The reaction was stirred at room temperature under H2 (1 atm) over night. The mixture was filtered through celite, and the recovered filtrate was concentrated to give 80 mg of crude product, that was further purified by HPLC on a Nucleodur 100-5 C18 (5 μm; 5 mm i.d. x 250 mm) with MeOH:H2O (65:35) as eluent (flow rate 1 mL/min), to give 60 mg of compound **7** (71%, tR= 10.0 min). [α]D25= +16.2 (*c* 0.07, CH3OH); selected 1H NMR (400 MHz, CD3OD): δH 3.64 (3H, s), 3.56 (1H, br s), 2.37 (1H, m), 2.25 (1H, m), 1.03 (3H, s), 0.94 (3H, d, *J* = 6.5 Hz), 0.70 (3H, s); 13C NMR (100 MHz, CD3OD): δC 174.6, 57.6, 57.4, 52.0, 50.1, 43.9, 41.3, 41.0, 37.5, 36.9, 36.6, 36.1, 32.2, 31.8, 30.4, 29.9, 29.1, 27.4, 27.0, 25.1, 24.2, 23.8, 22.0, 18.7, 12.4; HR ESIMS *m/z* 390.3378 [M + H]+, C25H44O2N requires 390.3372.

**Methyl 3β-ammino-5β-cholan-24-oic acid (6).** Compound **7** (20 mg, 51.4 x 10-3 mmol) was hydrolyzed with a methanol solution of sodium hydroxide (5%, 5 mL) in H2O (1 mL) for 8 h under reflux. The resulting solution was then concentrated under vacuum, diluted with water, acidified with HCl 6 N and extracted with ethyl acetate (3 x 50 mL). The collected organic phases were washed with brine, dried over Na2SO4 anhydrous and evaporated under reduced pressure. HPLC purification on a Nucleodur 100-5 C18 (5 μm; 5 mm i.d. x 250 mm) with MeOH/H2O (70:30) with 0.1% TFA as eluent (flow rate 1 mL/min), gave12 mg of **6** as white solid (62%, tR= 15.0 min). [α]D25= +10.0 (*c* 0.19, CH3OH); selected 1H NMR (400 MHz CD3OD): δH 3.58 (1H, br s), 2.30 (H, m), 2.21 (1H, m), 1.04 (3H, s), 0.95 (3H, d, *J* = 6.4 Hz), 0.71 (3H, s); 13C NMR (100 MHz, CD3OD): δC 170.6, 57.7, 57.5, 50.0, 43.9, 41.3, 41.0, 37.6, 36.9, 36.8, 36.1, 32.5, 30.5, 29.9, 29.2 (2C), 27.4, 27.0, 25.2, 24.3, 23.9, 22.0, 18.8, 12.5; HR ESIMS *m/z* 374.3064 [M - H]­, C24H40O2N requires 374.3059.

**Methyl 3β-ammino-5β-cholan-24-ol (8).** To a solution of methyl ester **7** (20 mg, 51.4 x 10-3 mmol) in dry THF (5 mL), at 0 °C dry methanol (15 μL, 0.36 mmol) and LiBH4 (180 μL, 2 M in THF, 0.36 mmol) was added. The resulting mixture was stirred for 1 h at 0 °C. The mixture was quenched by addition NaOH 1 M (10 μL and then ethyl acetate. The organic phase was washed with water, dried (Na2SO4) and concentrated. HPLC purification on a Nucleodur 100-5 C18 (5 μm; 10 mm i.d. x 250 mm) with MeOH/H2O (65:35) as eluent (flow rate 1 mL/min), gave 11 mg of compound **8** as a white solid (59%, tR= 7.5 min). [α]D25= +19.1 (*c* 0.07, CH3OH); selected 1H NMR (400 MHz CD3OD): δH 3.56 (1H, br s), 3.50 (2H, m), 2.28 (1H, m), 1.04 (3H, s), 0.95 (3H, d, *J* = 6.5 Hz), 0.71 (3H, s). 13C NMR (100 MHz CD3OD): δC 63.4, 57.7, 57.5, 50.0, 43.8, 41.4 (2C), 37.6, 36.9 (2C), 35.9, 33.1, 30.9, 30.2, 29.3, 27.5 (2C), 27.0, 25.2, 23.7, 23.1, 22.1, 19.0, 12.4; HR ESIMS *m/z* 362.3427 [M + H]+, C24H44ON requires 362.3423.

1H NMR (700 MHz, CD3OD) of compound **3**


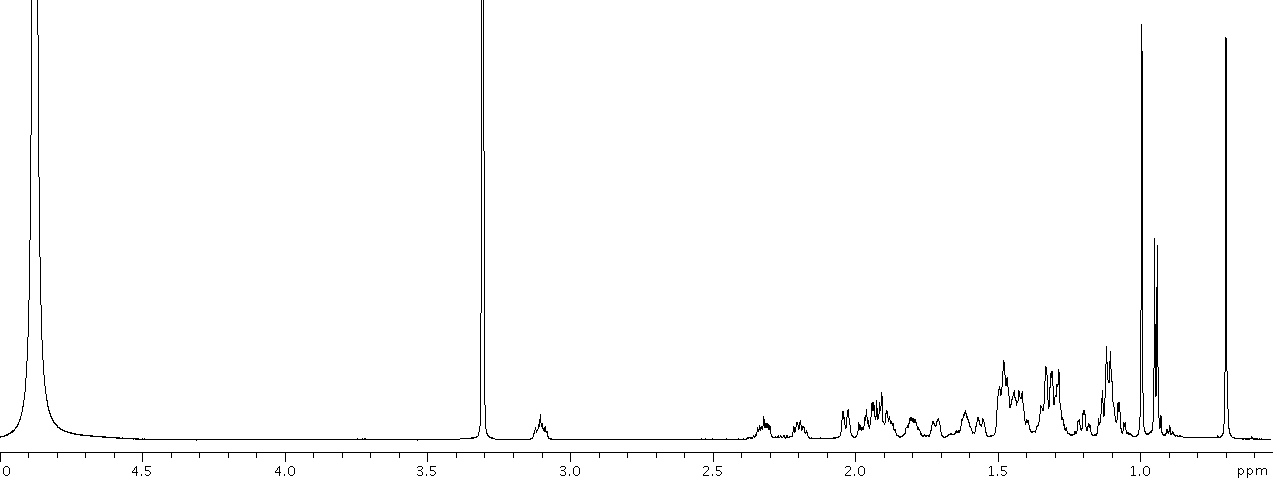


13C NMR (175 MHz, CD3OD) of compound **3**

**
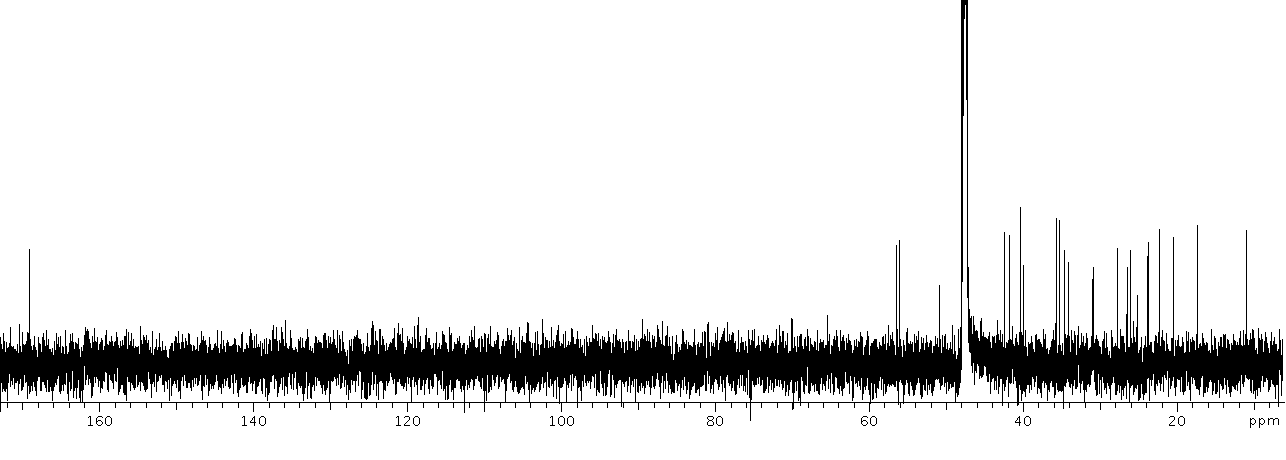
**

1H NMR (400 MHz, CD3OD) of compound **4**


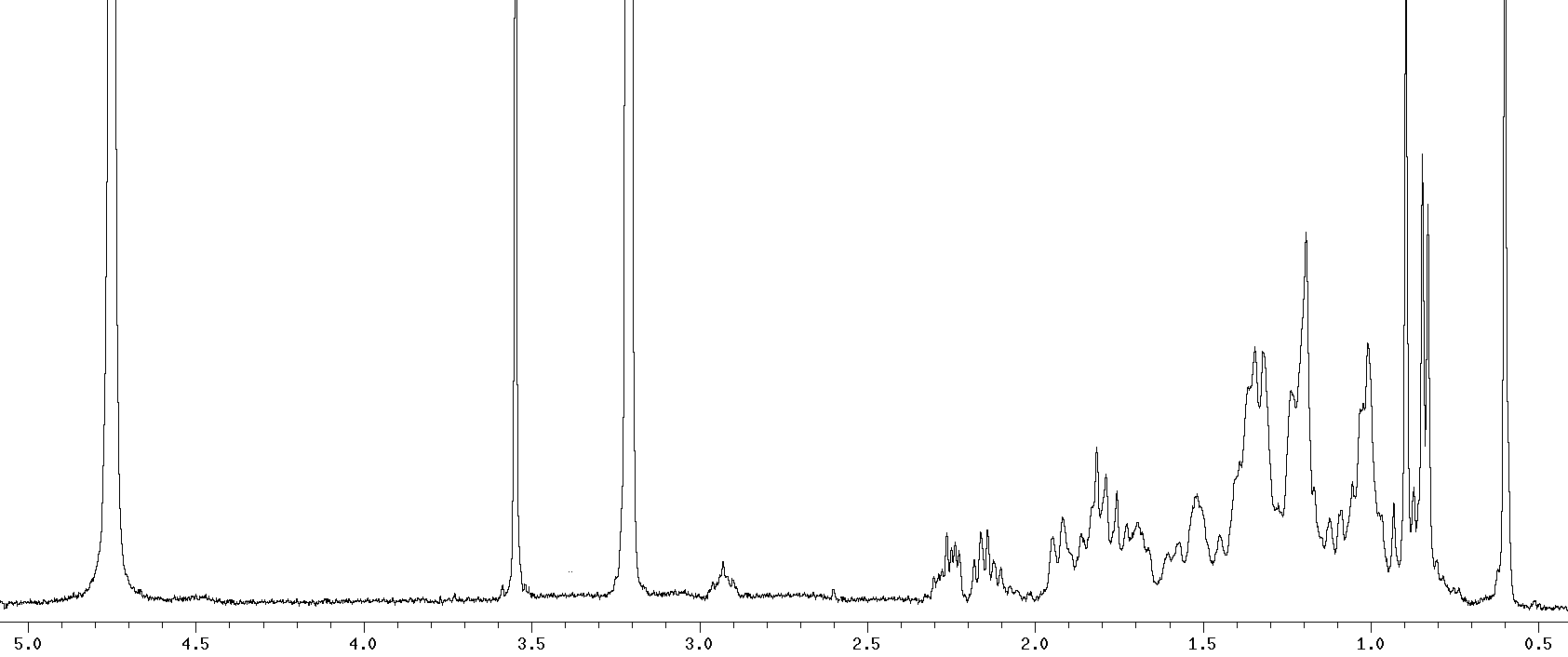


13C NMR (100 MHz, CD3OD) of compound **4**

1H NMR (700 MHz, CD3OD) of compound **5**


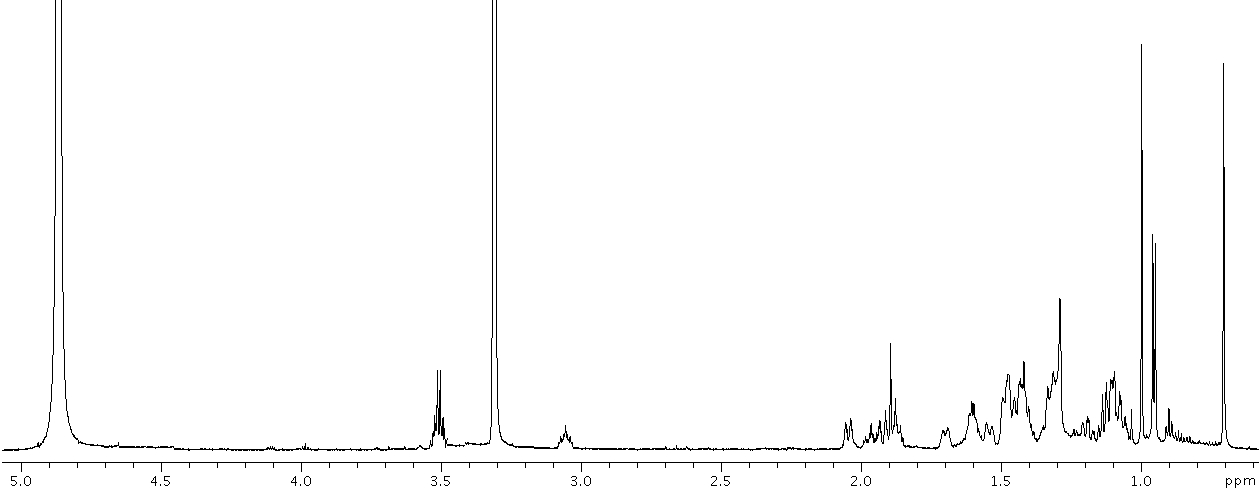


13C NMR (100 MHz, CD3OD) of compound **5**

**
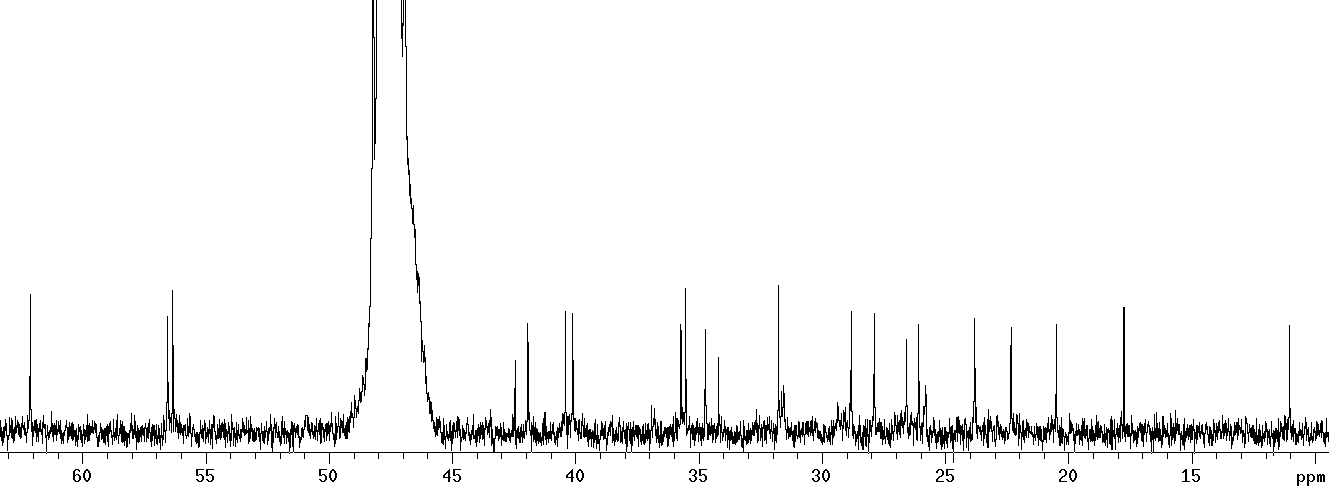
**

1H NMR (400 MHz, CD3OD) of compound **6**


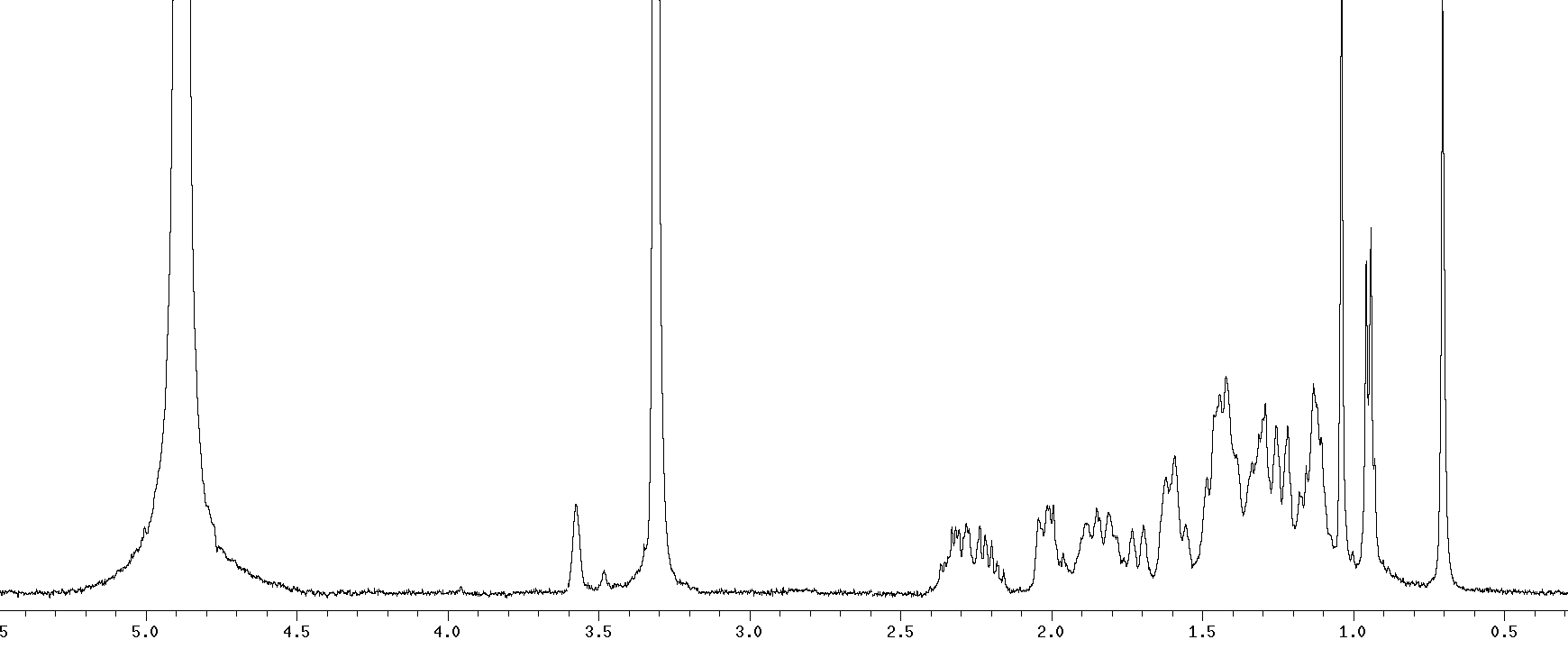


13C NMR (100 MHz, CD3OD) of compound **6**

**
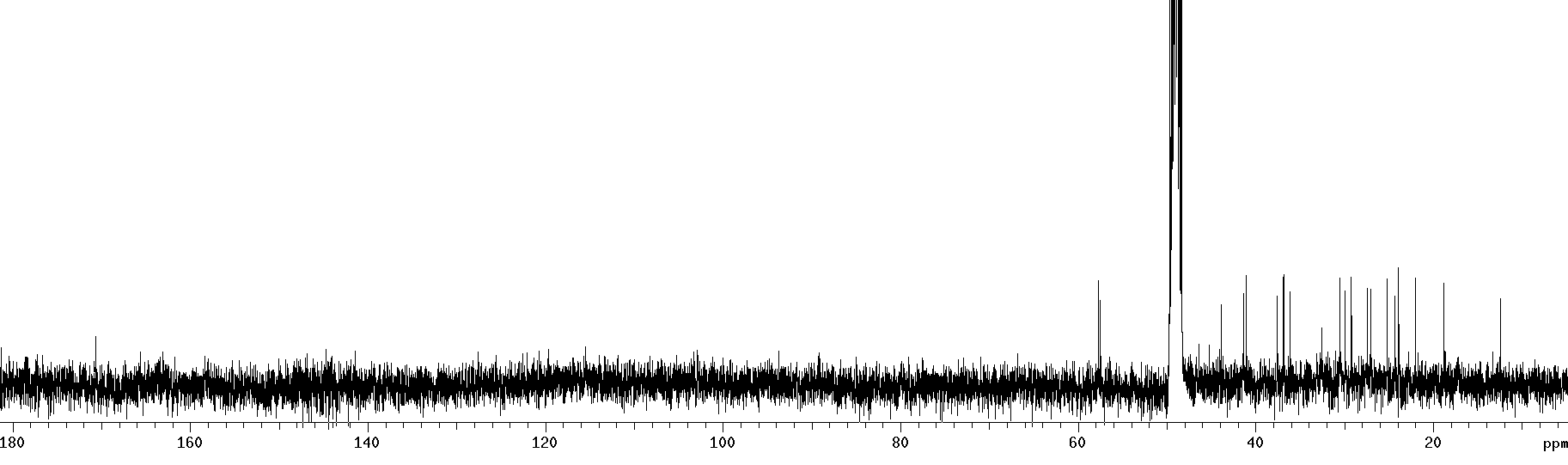
**

1H NMR (400 MHz, CD3OD) of compound **7**


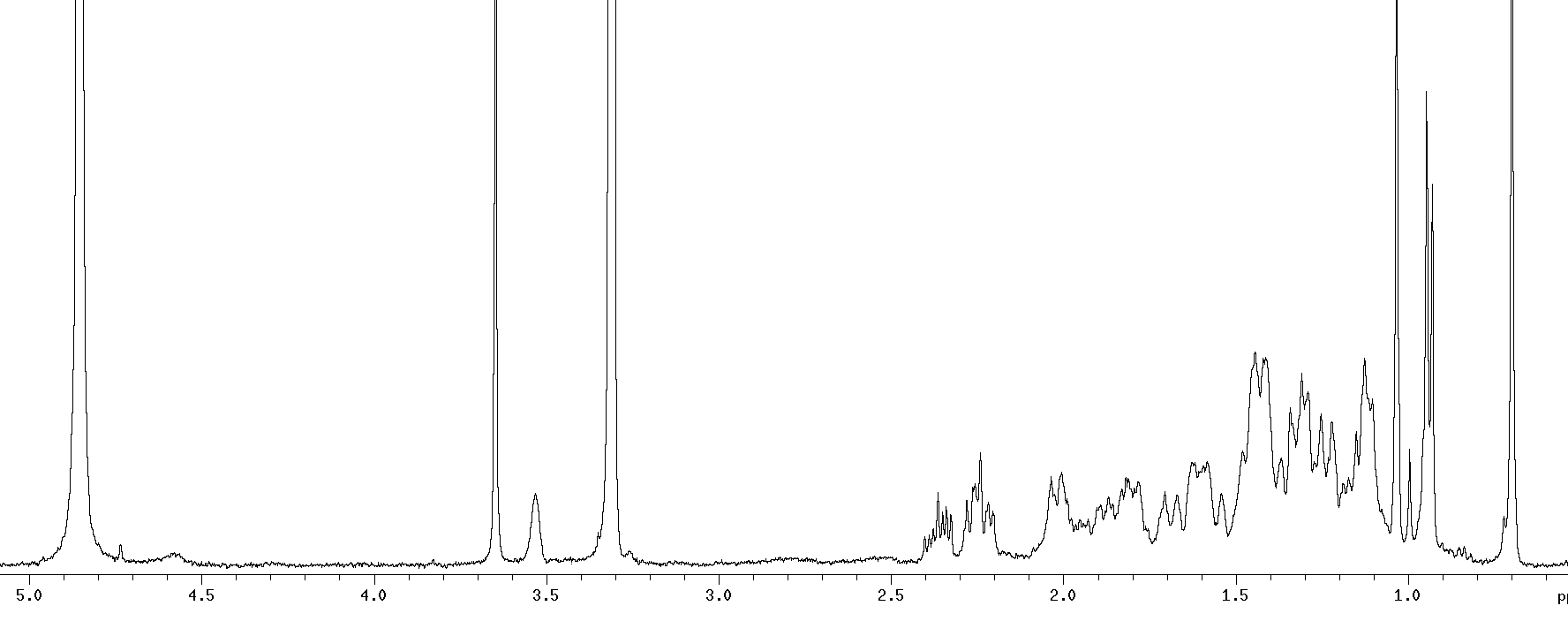


13C NMR (100 MHz, CD3OD) of compound **7**

**
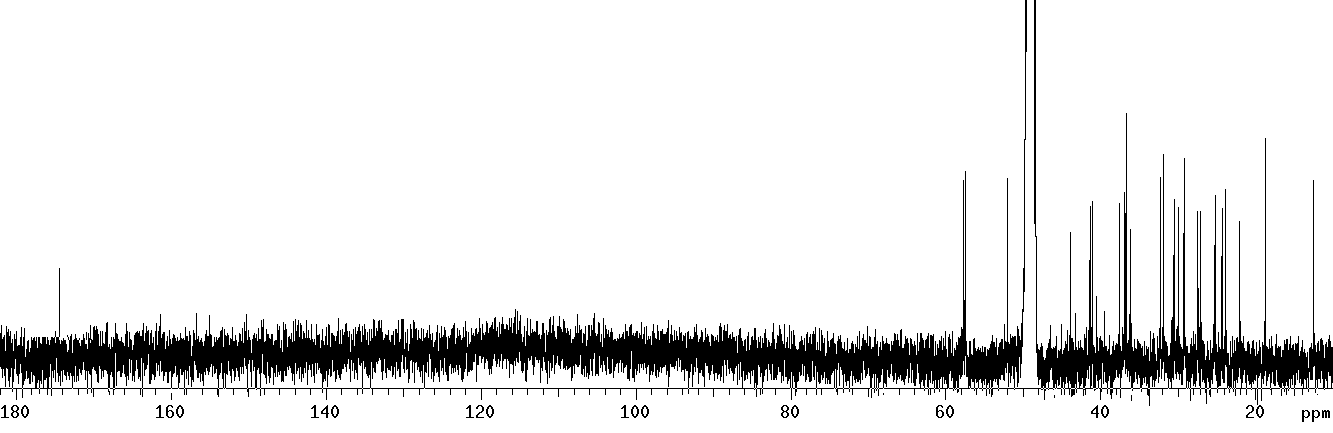
**

1H NMR (400 MHz, CD3OD) of compound **8**


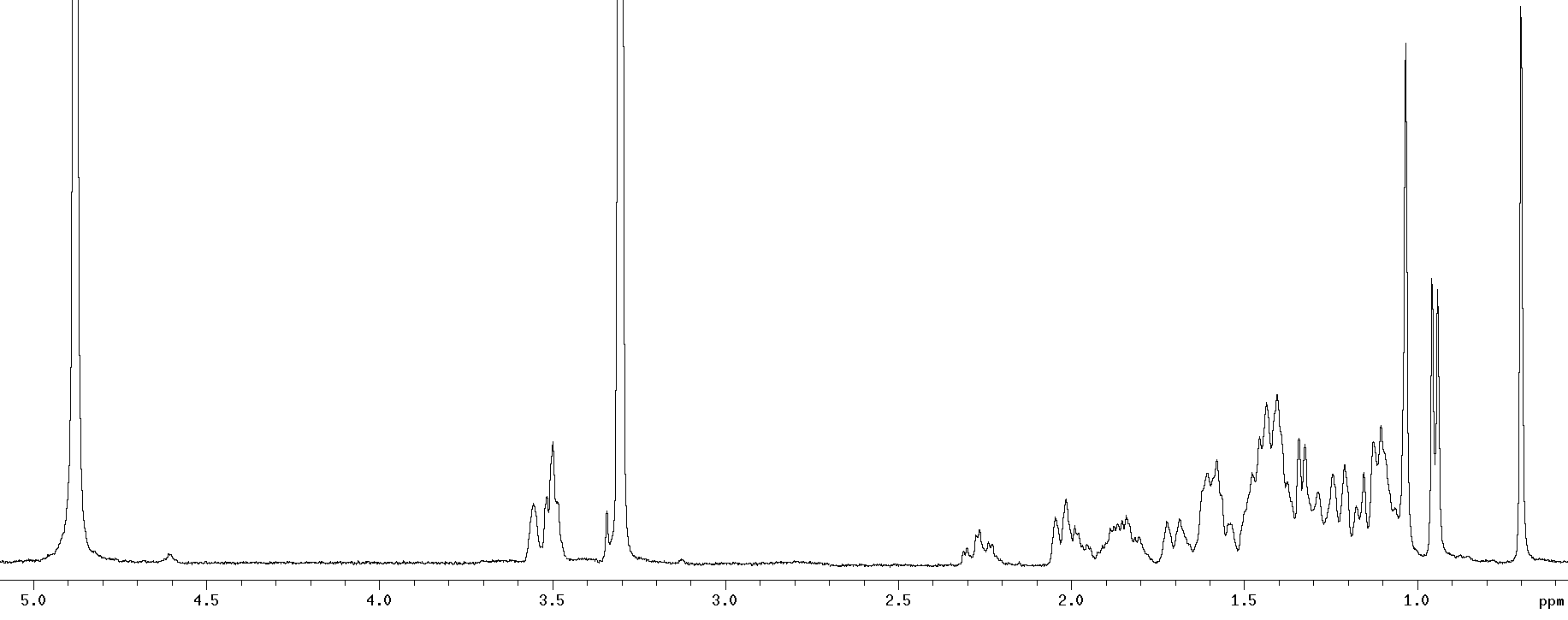


13C NMR (100 MHz, CD3OD) of compound **8**


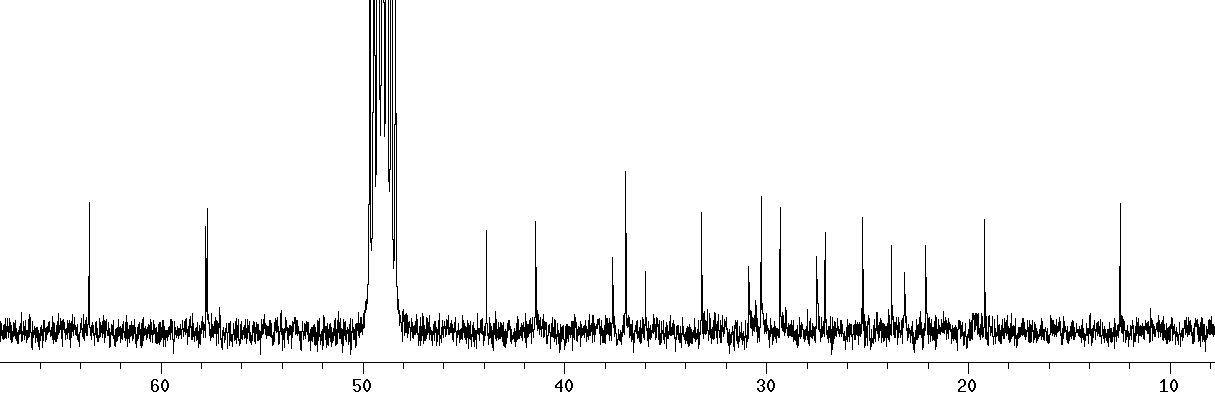


**Table S1.** Docking solutions and scores. The selected binding modes is highlighted in bold.

|  | **Solution** | **Score** |
| --- | --- | --- |
| **Glide** | **1** | **-6.928** |
|  | 2 | -6.833 |
|  | 3 | -6.787 |
|  | 4 | -6.546 |
|  | 5 | -6.531 |
